# Structural Analysis of the Gd–Au–Al 1/1 Quasicrystal Approximant Phase across Its Composition-Driven Magnetic Property Changes

**DOI:** 10.1021/acs.inorgchem.3c01967

**Published:** 2023-08-29

**Authors:** Yu-Chin Huang, Ulrich Häussermann, Girma H. Gebresenbut, Fernand Denoel, Cesar Pay Gómez

**Affiliations:** † Department of Chemistry, Ångström Laboratory, Uppsala University, 751 21 Uppsala, Sweden; ‡ Department of Materials and Environmental Chemistry, Stockholm University, 106 91 Stockholm, Sweden; § Department of Materials Science and Engineering, Uppsala University, Box 35, 751 03 Uppsala, Sweden

## Abstract

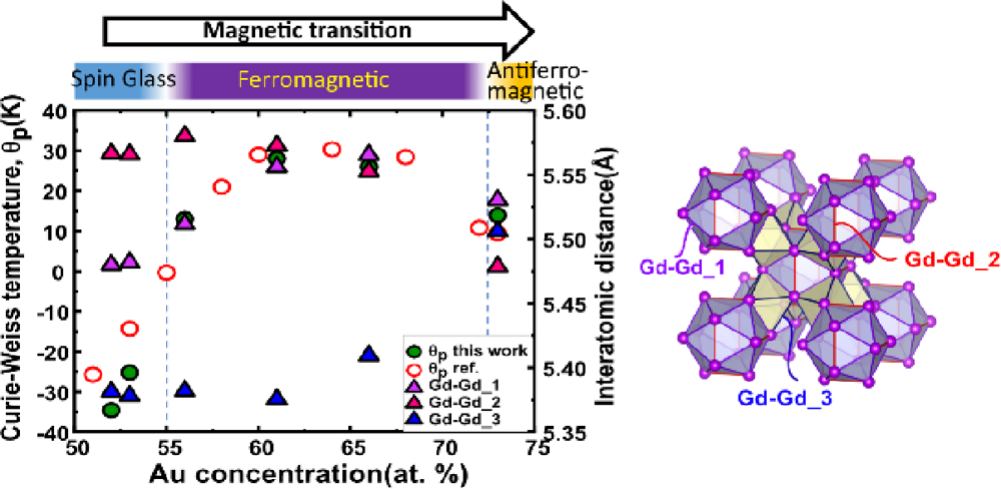

Gd_14_Au_*x*_Al_86–*x*_ Tsai-type 1/1 quasicrystal approximants (ACs) exhibit three magnetic orders that can be finely tuned by the valence electron concentration (*e*/*a* ratio). This parameter has been considered to be crucial for controlling the long-range magnetic order in quasicrystals (QCs) and ACs. However, the nonlinear trend of the lattice parameter as a function of Au concentration suggests that Gd_14_Au_*x*_Al_86–*x*_ 1/1 ACs are not following a conventional solid solution behavior. We investigated Gd_14_Au_*x*_Al_86–*x*_ samples with *x* values of 52, 53, 56, 61, 66, and 73 by single-crystal X-ray diffraction. Our analysis reveals that increasing Au/Al ordering with increasing *x* leads to distortions in the icosahedral shell built of the Gd atoms and that trends observed in the interatomic Gd–Gd distances closely correlate with the magnetic property changes across different *x* values. Our results demonstrate that the e/a ratio alone may be an oversimplified concept for investigating the long-range magnetic order in 1/1 ACs and QCs and that the mixing behavior of the nonmagnetic elements Au and Al plays a significant role in influencing the magnetic behavior of the Gd_14_Au_*x*_Al_86–*x*_ 1/1 AC system. These findings will contribute to improved understanding towards tailoring magnetic properties in emerging materials.

## Abstract 2

We conducted a re-examination of six Gd_14_Au_*x*_Al_86−*x*_ 1/1 quasicrystal approximants (ACs) with varying Au concentration using single-crystal X-ray diffraction and magnetic susceptibility measurements. Our analysis revealed distortions in the icosahedral shell comprised of Gd atoms, and we also observed that trends in the interatomic Gd−Gd distances closely correlate with the Curie−Weiss temperature variations across different Au concentrations. In this paper, we discuss the changes in magnetic behavior in Gd_14_Au_*x*_Al_86−*x*_ 1/1 ACs from a structural perspective.

## Introduction

The magnetic properties of intermetallic icosahedral quasicrystals (QCs) have attracted considerable attention because of expectations about unique magnetic states based on the long-range ordering of spins in a quasiperiodic structure.^1,2^ Recent focus has been especially on Tsai-type QCs and related approximant crystal (AC) phases containing rare-earth (R) elements and exploring the systems R–Au–Al, R–Au–Ga, and R–Au–Si (R = Ce, Gd, Tb, Dy, Ho, and Tm).^1,3−6^ Whereas the stability of QCs is assumed to be closely linked to the valence electron concentration (i.e., number of valence electron per atom (*e*/*a*))—which for Tsai-type QCs appears to be restricted to the narrow range of 2–2.15—AC phases are found much more frequently in intermetallic systems and with a wider range of *e*/*a* (1.75–2).^7^

**Figure 1:**
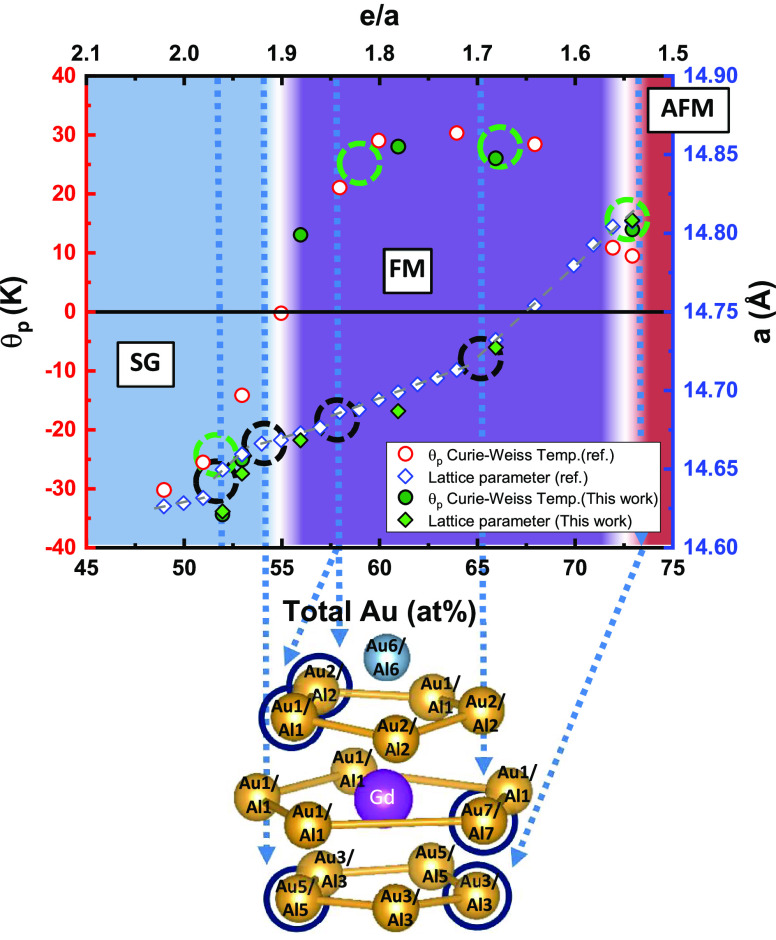
Paramagnetic Curie–Weiss temperatures (θ_p_, circle icon) and lattice parameters (diamond icon) across as a function of Au concentration for Gd_14_Au_*x*_Al_86–*x*_ 1/1 AC phases. The background coloring highlights the different magnetic regimes, from SG to FM and AFM, which was adopted from Ishikawa et al. (2018).^4^ The green color icons are the data from this work, while the white color icons are the reference data from Ishikawa et al. (2016).^3^ The black dotted circles show the inflection points in the trend of the lattice parameter, and the green dotted circles indicated discontinuities in the trend of the Curie–Weiss temperature. The lower part of the figure shows the coordination of Gd atoms by 16 Au and Al atoms in the shape of a monocapped, double pentagonal antiprism. Each inflection point, apart from the first one, links to an occupancy saturation event in the different atomic positions of the 1/1AC structure (blue dashed lines). Adapted with permission from refs (3) and (4). Copyright 2016 and 2023 American Physical Society.

A particularly exciting system is Gd–Au–Al for which a QC is not known but the 1/1 AC phase Gd_14_Au_*x*_Al_86–*x*_ with an extraordinarily large range of composition, *x* = 49–72, corresponding to the *e*/*a* range of 2.02–1.56.^3^ With increasing Au concentration (decreasing *e*/*a*), magnetism for Gd_14_Au_*x*_Al_86–*x*_ changes from spin glass (SG) behavior (*e*/*a* > 1.9) to ferromagnetic (FM, 1.57 < *e*/*a* < 1.9) to antiferromagnetic (AFM, *e*/*a* < 1.57)^3,4^ (Figure 1). In other words, if the rare-earth element is considered as fully occupied across the homogeneity range of Gd_14_Au_*x*_Al_86–*x*_, the ratio of the nonmagnetic elements Au and Al would drive the magnetic property changes. A similar observation has been recently reported for the quaternary Gd_14_(Ga,Pd,Au)_86_ system for which (more limited) *e*/*a* variations of 1.92 to 1.74 lead to transition from SG to FM.^8^ Using a minimal magnetic model including only RKKY interaction, Miyazaki et al. could show from Monte Carlo simulations that the change of Au/Al ratio for Gd_14_Au_*x*_Al_86–*x*_ correlates with a change in the Fermi wavenumber, driving consecutive magnetic transitions.^9^ Yet there are some inconsistencies between experimentally observed and simulated results.

If *e*/*a* is solely responsible for magnetic property changes, one would expect that Gd_14_Au_*x*_Al_86–*x*_ represents an ideal alloy and, in this case, its cubic unit cell volume (or lattice parameter *a*) should follow Vegard’s law. Figure 1 includes the lattice parameter changes across the homogeneity range. It is clear that the overall trend deviates from a linear Vegard-like behavior. At least the different magnetic regions would have somewhat different slopes. Thus, one could suspect that the magnetic property changes are accompanied also by small crystal structural changes, and elucidating the detailed crystal structure of Gd_14_Au_*x*_Al_86–*x*_ may help in a deeper understanding of the magnetic property changes.

Ishikawa et al. prepared Gd_14_Au_*x*_Al_86–*x*_ materials by stoichiometric arc-melting of elemental mixtures, followed by annealing at 800 °C (for 50 h)^3^ or by remelting in an induction furnace.^4^ While this guarantees compositionally homogeneous samples, detailed structural information has not been extracted (or has not been possible to extract) from powder X-ray diffraction data. So far, Gd_14_Au_73_Al_13_^4^ is the only one reported sample with a single-crystal refinement result. In this work, we prepared phase-pure and compositionally homogeneous single-crystal samples of Gd_14_Au_*x*_Al_86–*x*_ from Gd-poor reaction mixtures through solidification from the melt. The liquidus line of the Al–Au phase diagram^10^ is at relatively low temperatures (between 550 and 650 °C) for the investigated compositional range of Gd_14_Au_*x*_Al_86–*x*_, which suggests that the AC phase can be precipitated from Gd-poor melts Gd_*y*_(Au_*z*_Al_100–*z*_)_100–*y*_ (*z* = 50–82 and *y* < 10). We performed structural studies by single-crystal X-ray diffraction (SCXRD) on high-quality single crystals from melt-precipitated Gd_14_Au_*x*_Al_86–*x*_ samples with the goal of identifying potential patterns from the interatomic distance distributions that may correlate with the magnetic behavior change.

## Experimental Section

Granules of Gd (99.999%), Au (99.99%), and Al (99.9999%) were acquired from Chempur and used as starting materials. Prior to the synthesis reactions, Au and Al were arc-melted in various ratios of Au_*z*_Al_100–*z*_ (*z* = 50, 55, 60, 67, 74, and 82) to produce (inhomogeneous) melts. The actual reaction syntheses were then obtained from a mixture of Gd and the aforementioned alloy Au_*z*_Al_100–*z*_ with a molar ratio of 1:11.5, i.e., Gd_8_(Au_*z*_Al_100–*z*_)_92_. The employed reaction mixtures are abbreviated as GdAA(*z*); see Table 1. Reaction mixtures were investigated with differential scanning calorimetry (DSC) prior to the solution-growth synthesis to extract the liquidus temperatures for the ternary compositions.^5^ To synthesize the 1/1 AC phase samples, 2 g of the precursor mixture was loaded inside an Ar-filled glovebox into an alumina crucible being part of a Canfield crucible set (CCS)^11^ purchased from LSP Industrial Ceramics (USA), which was subsequently sealed in a stainless-steel ampule. The ampules were placed inside a silica wool-insulated stainless-steel cylinder, then heated in a commercial multistep programmable muffle furnace to 1100 °C, and dwelled at that temperature for 5 h to achieve homogeneous melts. Finally, the temperature was lowered to a target temperature between 600 and 900 °C (cf. Table 1) at a rate of 2 °C/h. Once the target temperature was reached, the precipitated 1/1 AC phase was equilibrated with the melt for 24 h, after which the crystalline AC phase was separated from the melt by isothermal centrifugation (in the Supporting Information, Figure S2).

**Table 1:** Nominal Compositions and EDX Results

sample	nominal composition	reactants	formation temp. (°C)	centrifuged temp. (°C)	EDX comp.
GdAA(50)	Gd_8_(Au_50_Al_50_)_92_	Gd + Au_50_Al_50_	930	∼750	Gd_14.0(2)_Au_52.0(3)_Al_34.0(4)_
GdAA(55)	Gd_8_(Au_55_Al_45_)_92_	Gd + Au_55_Al_45_	940	∼900	Gd_14.0(2)_Au_53.0(2)_Al_33.0(2)_
GdAA(60)	Gd_8_(Au_60_Al_40_)_92_	Gd + Au_60_Al_40_	941	∼600	Gd_13.9(4)_Au_56.0(3)_Al_30.2(5)_
GdAA(67)	Gd_8_(Au_67_Al_33_)_92_	Gd + Au_67_Al_33_	909	∼600	Gd_13.8(4)_Au_61.4(3)_Al_24.8(6)_
GdAA(74)	Gd_8_(Au_74_Al_26_)_92_	Gd + Au_74_Al_26_	870	∼600	Gd_13.6(3)_Au_66.4(4)_Al_20.0(4)_
GdAA(82)	Gd_8_(Au_82_Al_18_)_92_	Gd + Au_82_Al_18_	800	∼800	Gd_13.6(2)_Au_73.1(6)_Al_13.3(6)_

DSC measurements were performed with a NETZSCH STA 449 F1 Jupiter instrument and using polycrystalline sapphire crucibles (OD = 5 mm; ID = 4 mm). To extract the liquidus temperatures (crystallization temperatures from the melt), 200 mg of Gd_8_(Au_*z*_Al_100–*z*_)_92_ reaction mixture (nominal composition) was subjected to two heating/cooling cycles to 1150 °C. The heating/cooling rates of the first and second cycles were 25 and 10 °C/min, respectively. To analyze the melt-centrifuged single AC phase, sample specimens (typically faceted grains) with a total mass of ∼50 mg were subjected to a heating/cooling cycle to 1150 °C at a rate of 10 °C/min. All measurements were performed under a constant flow of Ar at 40 mL/min, and the presence of an oxygen getter and an empty crucible served as the reference. All the DSC data can be found in the Supporting Information, Figures S1 and S5, respectively.

Powder X-ray diffraction (PXRD) data were collected on a Bruker D8-ADVANCE diffractometer with θ – 2θ diffraction geometry and Cu–Kα radiation (Kα1 = 1.540598 Å and Kα2 = 1.544390 Å) at room temperature. Powdered samples were applied to a zero-diffraction plate, and the diffraction patterns were measured with an internal silicon standard for the 2θ range of 10–90° with a step size of 0.01°and an exposure time of 1 s. The analysis of PXRD data was performed using HighScore Plus 3.0 software from PANalytical,^12^ and the lattice parameters were indexed and refined with the Checkcell program package.^13^

To collect high-quality single-crystal X-ray diffraction (SCXRD) data, we selected faceted millimeter-sized single grain crystals and removed the residual flux from the surface by cutting them into smaller grains with a size of 50–100 μm to reduce sample absorption effects during SCXRD measurements. All of the SCXRD measurements were performed at room temperature on a D8 venture single-crystal X-ray diffractometer with a Mo-sealed tube X-ray source (Mo Kα = 0.71073 Å) and a shutterless PHOTON 100 CMOS detector. To achieve optimal data quality, data were collected with 40 s of exposure time and a step size of 0.3°. SCXRD data sets were processed using Apex III software^14^ for integration, absorption correction, and data reduction. Structure refinements were done with the Jana 2006 package.^15^ The electron densities were calculated from observed SCXRD intensities by the Fourier method and visualized in VESTA 3.5.8.^16^

Samples for compositional analysis were prepared by cross-sectional polishing using an Ar ion beam in a JEOL IB-09010CP Cross-section polisher. The compositional analysis was performed in a Zeiss LEO 1550 scanning electron microscope with Oxford Aztec Energy Dispersive X-ray spectroscopy (EDX), equipped with an 80 mm^2^ Silicon Drift Detector. The compositional homogeneity was examined through EDX data collected with an acceleration voltage of 20 kV and high current with 6.5 mm working distance for several selected areas (∼100 × 100 μm) on at least 10 points for each sample.

The magnetic susceptibility measurements were performed in an MPMS XL SQUID magnetometer from Quantum Design Inc. Zero-field cooled (ZFC) and field cooled (FC) dc magnetic susceptibility was acquired at low temperature at a fixed field of 10 Oe in the range of 2–40 K, covering the magnetic transitions of the samples (spin freezing, *T*_f_; Curie, *T*_c_; Néel temperature, *T*_N_). The Curie–Weiss parameters θ_p_ were extracted from magnetic susceptibility data performed under 5000 Oe between 2 and 300 K at 1 K/min. All of the PXRD and magnetic susceptibility data can be found in the Supporting Information.

## Results and Discussion

### Synthesis and Characterization

The six reaction mixtures GdAA(*z*) (cf. Table 1) were intended to cover the compositional range of the cubic 1/1 AC Gd_14_Au_*x*_Al_86–*x*_ phase, in particular within the different magnetic ranges correlating with the different slopes in the lattice parameter plot as a function of the total Au concentration (cf. Figure 1). The Au_*z*_Al_100–*z*_ melts have a sharply increased liquidus temperature for *z* < 50 and *z* > 82.^10^ The synthesis of approximants Gd_14_Au_*x*_Al_86–*x*_ from melts of composition Gd_8_(Au_*z*_Al_100–*z*_)_92_ assumes a pseudobinary behavior Gd–(Au_*z*_Al_100–*z*_), and we expected that the liquidus temperature for the ternary system correlates with that of binary Au_*z*_Al_100–*z*_. According to the DSC investigation of GdAA(*z*) (cf. Figure S1 in the Supporting Information), the liquidus temperatures are in the range of 800–950 °C for 50 < *z* < 82. These temperatures are listed in Table 1.

In practice, the synthesis of 1/1 AC Gd_14_Au_*x*_Al_86–*x*_ samples by the melt precipitation method succeeds only in the range of 53 < *x* < 73 wherein single-phase, millimeter-sized, faceted grains could be readily isolated from the melt (see the Supporting Information, Figure S2). Higher *x* values will still allow for reasonably low liquidus temperatures for the ternary system. However, at *x* > 73, the formation of an orthorhombic phase with composition GdAu_6.75−δ_Al_0.5+δ_ (δ ≈ 0.54) is preferred, which we reported elsewhere.^17^ For *x* < 53, liquidus temperatures become too high and melts too viscous for practical handling of the centrifugation separation. Nevertheless, we included GdAA(50) in this investigation, although melt separation was not satisfactory, i.e, the centrifuged product contained significant amounts of other phases. Interestingly, in addition to crystals of the regular 1/1 AC Gd_14_Au_*x*_Al_86–*x*_ phase, we could also identify a closely related superstructure phase (see Figure S3), which we will report on in a forthcoming publication.

The actual composition of the obtained 1/1 AC Gd_14_Au_*x*_Al_86–*x*_ phase (as determined from EDX analysis, cf. Table 1) deviates, sometimes substantially, from the nominal Au/Al ratio of the reaction mixture. This indicates that a pseudobinary behavior Gd–(Au_*z*_Al_100–*z*_) is not followed. However, the actual composition of the samples, *x* = 52 (GdAA(50)), *x* = 53 (GdAA(55)), *x* = 56 (GdAA(60)), *x* = 61 (GdAA(67)), *x* = 66 (GdAA(74)), and *x* = 73 (GdAA(82)), still distributes meaningfully across the homogeneity range of Gd_14_Au_*x*_Al_86–*x*_. Figure S4 shows the PXRD patterns of the obtained Gd_14_Au_*x*_Al_86–*x*_ products. The refined lattice parameters from the PXRD pattern are reported in Table 2 and included together with results from the previous work of Ishikawa et al.^3^ in Figure 1. We note a rather close agreement with the previous work and thus confirm the discontinuous trend of the lattice parameter expansion with increasing *x*. In parallel, peak broadening was observed with increasing Au content for which we currently have no explanation. The thermally annealed samples by Ishikawa et al. with similar compositions do not show peak broadening.^3^

According to DSC measurements, the peritectic decomposition (or melting) of 1/1 AC Gd_14_Au_*x*_Al_86–*x*_ samples is between 900 and 1050 °C, cf Figure S5, Supporting Information. We also measured the magnetic susceptibilities of our samples and found good agreement with the previous studies.^3,4^ The measurement results are listed in Figure S6 and Table S1 and are also included in Figures 1 and 4b.

### 1/1 AC Crystal Structure and Refinement of Gd_14_Au_*x*_Al_86–*x*_

**Figure 2:**
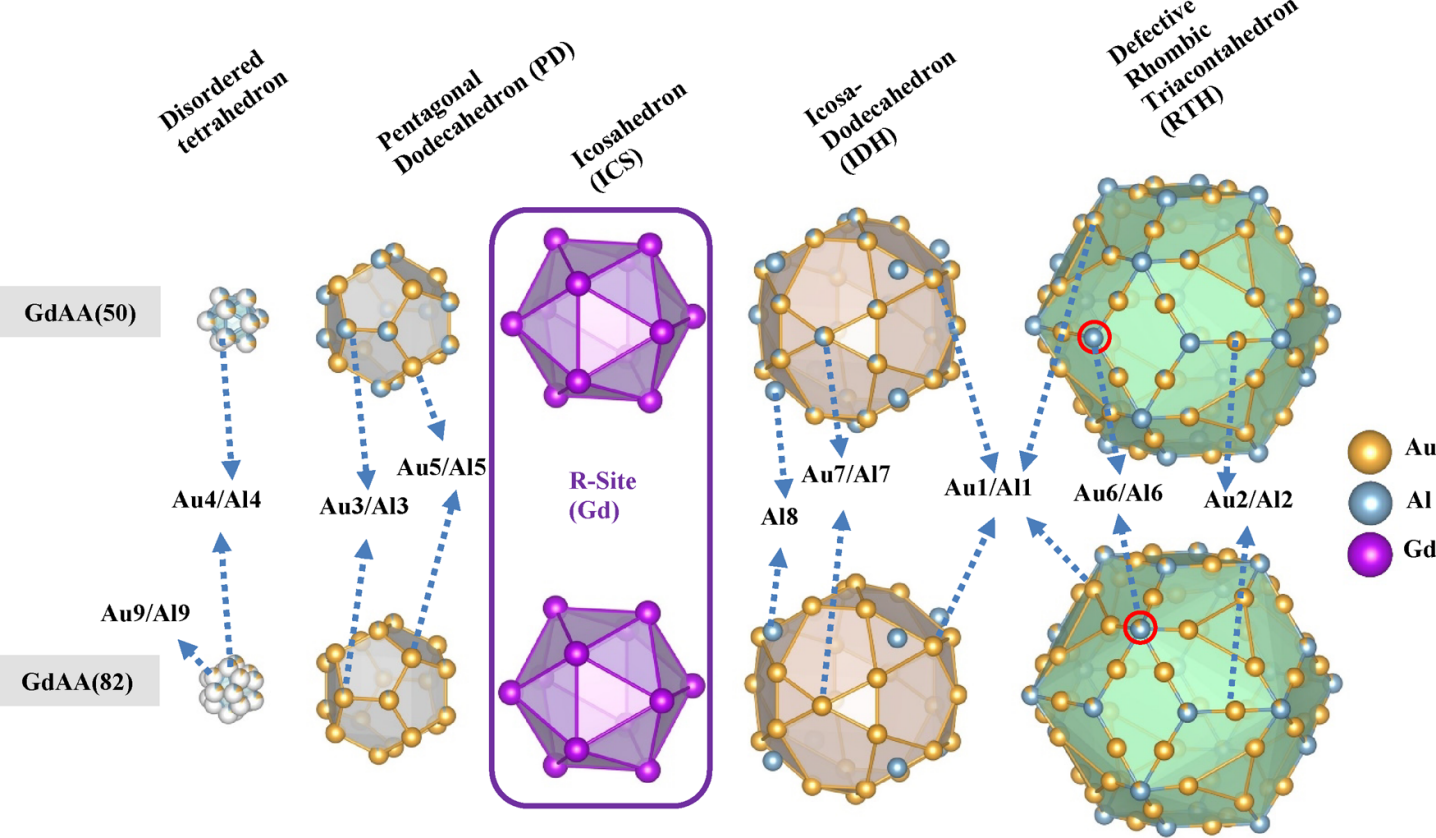
Comparison of the center and shells (PD, ICS, IDH, and RTH) of the Tsai cluster for Gd_14_Au_*x*_Al_86–*x*_ with refined compositions Gd_14_Au_52_Al_34_ (sample GdAA(50)) and Gd_14_Au_73_Al_13_ (sample GdAA(82)). The latter sample has the highest Au concentration and differs from the other samples by having an extra position (Au9/Al9) for describing the disordered tetrahedron at the cluster center and by the fact that the Al6 site in the RTH shell becomes mixed with Au (marked with a red circle).

The structure of the Gd_14_Au_*x*_Al_86–*x*_ AC is that of the conventional cubic 1/1 Tsai-type AC with the centrosymmetric space group *Im*3̅ (no. 204). Its crystallographic composition is Gd(Au,Al)_6.333_ (the normalized composition is Gd_13.6_(Au,Al)_86.4_).^18,19^ The structure is usually conveniently described as a bcc packing of (Tsai) clusters consisting of concentric shells,^20^ shown in Figure 2.

The first shell corresponds to a pentagonal dodecahedron (PD) that is defined by the positions Au3/Al3 (24g) and Au5/Al5 (16f). The next shell is an icosahedron (ICS) consisting of the rare-earth atoms RE(Gd), position Gd1 (24g). The third shell is a 30 vertex icosidodecahedron (IDH), defined by Au1/Al1 and Au7/Al7 atoms (positions 48h and 12d, respectively). The outermost shell corresponds to a rhombic triacontahedron (RTH) that involves the positions Au6/Al6(12e), Au2/Al2 (24g), and Au1/Al1 (48h), the last of which is shared with the IDH shell (cf. Figure 2). The position Al8 (8c) is situated between the IDH and RTH shells and located on the 3-fold rotational axes. Clusters are connected along the [100] direction by sharing common rhomboid faces defined by the Au6–Al6 position and by interpenetrating along the [111] direction. Finally, Tsai clusters in Gd_14_Au_*x*_Al_86–*x*_ are centered by (Au,Al)_4_ tetrahedra (position Au4/Al4), which at room temperature are orientationally disordered. Crystallographically, the centering tetrahedra are described by a site 24g, with a constrained occupancy of 1/3, which emulates the disorder as three superimposed orientations.

Structure refinement results are summarized in Table 2, with refined occupancies and atomic positions listed in Table 3. The values for the extracted lattice parameters at different Au concentrations are included in Figure 1. The refined compositions of the crystals correspond well to the EDX results. The GOF and *wR*_2_ are located in the ranges of 1.51–2.30 and 0.0624–0.0788, respectively (cf. Table 2), which implies that the structure refinement is reliable.

**Table 2:** Structure Refinement Results

sample	GdAA(50)	GdAA(55)	GdAA(60)	GdAA(67)	GdAA(74)	GdAA(82)
ref. comp.	Gd_13.6_Au_50.8(3)_Al_35.6(3)_	Gd_13.6_Au_51.2(3)_Al_35.2(3)_	Gd_13.6_Au_54.5(2)_Al_31.9(2)_	Gd_13.6_Au_60.5(2)_Al_25.9(2)_	Gd_13.6_Au_65.9(1)_Al_20.5(1)_	Gd_13.6_Au_73.8(2)_Al_12.5(2)_
EDX comp.	Gd_14.0(2)_Au_52.0(3)_Al_34.0(4)_	Gd_14.0(2)_Au_53.0(2)_Al_33.0(2)_	Gd_13.9(4)_Au_56.0(3)_Al_30.2(5)_	Gd_13.8(4)_Au_61.4(3)_Al_24.8(6)_	Gd_13.6(3)_Au_66.4(4)_Al_20.0(4)_	Gd_13.6(2)_Au_73.1(6)_Al_13.3(6)_
molar mass (g/mol)	960.5	984.3	1007.4	1081.8	1149.3	1241.6
temp. of meas. (°C)	20	20	20	20	20	20
space group	*Im*3̅ (204)	*Im*3̅ (204)	*Im*3̅ (204)	*Im*3̅ (204)	*Im*3̅ (204)	*Im*3̅ (204)
*a* axis (Å) (SCXRD)	14.6382(4)	14.6345(2)	14.6694(1)	14.6875(6)	14.7314(2)	14.8034(1)
*a* axis (Å) (PXRD)	14.6447(8)	14.6464(2)	14.6677(4)	14.6863(7)	14.7268(2)	14.8079(9)
cell volume (Å^3^)	3136.63(15)	3134.25(7)	3156.7(4)	3168.4(2)	3196.92(8)	3244.0(5)
*Z*	24	24	24	24	24	24
calc. density (g/cm^3^)	12.2034	12.2862	12.718	13.6097	14.3275	15.2537
abs. coeff. (mm^–1^)	116.822	117.855	123.689	135.294	144.871	157.493
indep. reflections	892	888	1447	1442	1453	1565
obs. reflections	33577	29194	37931	30200	29907	20806
*R*_int_ (obs/all)	7.20/7.30	5.21/5.28	6.79/6.89	8.95/9.26	9.09/9.49	7.38/8.00
refined parameters	93	93	92	90	90	103
redundancy	37.642	32.876	26.214	20.943	20.53	13.295
*R*_1_ (obs/all)	0.0266/0.0298	0.0241/0.0270	0.0398/0.0485	0.0360/0.0475	0.0337/0.0468	0.0402/0.0656
*wR*_2_ (obs/all)	0.0657/0.0665	0.0624/0.0632	0.0788/0.0805	0.0776/0.0802	0.0656/0.0691	0.0749/0.0800
GOF on *F*^2^ (obs/all)	2.07/2.13	2.15/2.20	2.25/2.35	1.83/1.93	1.51/1.58	1.60/1.76
Δρ_max′_Δρ_min′_ (e/A^3^)	5.04/–2.44	4.76/–2.67	8.57/–6.57	5.61/–5.91	6.10/–5.75	6.22/–6.99

**Table 3:** Refined Occupancies and Atomic Positions

**atom**	**Wyck.**	**compound**	**S.O.F.**	***x*/*a***	***y*/*b***	***z*/*c***	***U***_**eq**_
Gd1	24g	GdAA(50)	1	0.19014(4)	0.30233(4)	0	0.01117(2)
		GdAA(55)	1	0.19017(4)	0.30254(4)	0	0.01578(2)
		GdAA(60)	1	0.19020(3)	0.30356(3)	0	0.01358(1)
		GdAA(67)	1	0.18971(4)	0.30595(4)	0	0.00474(1)
		GdAA(74)	1	0.19428(3)	0.31154(3)	0	0.00756(1)
		GdAA(82)	1	0.19746(5)	0.31501(5)	0	0.01091(2)
Au1/Al1	48h	GdAA(50)	0.783(3)/0.217(3)	0.34280(3)	0.19542(3)	0.10612(3)	0.01984(2)
		GdAA(55)	0.798(3)/0.202(3)	0.34268(3)	0.19552(3)	0.10602(3)	0.02450(1)
		GdAA(60)	0.884(2)/0.116(2)	0.34240(2)	0.19637(2)	0.10527(2)	0.02030(1)
		GdAA(67)	1/0	0.34075(2)	0.19898(2)	0.10407(2)	0.00744(9)
		GdAA(74)	1/0	0.34196(2)	0.19707(2)	0.10547(2)	0.01037(8)
		GdAA(82)	1/0	0.34545(3)	0.20072(3)	0.10808(3)	0.01475(1)
Au2/Al2	24g	GdAA(50)	0.854(4)/0.145(4)	0.5	0.09478(4)	0.14805(4)	0.02148(2)
		GdAA(55)	0.860(4)/0.140(4)	0.5	0.09469(4)	0.14799(4)	0.02572(2)
		GdAA(60)	0.872(3)/0.128(3)	0.5	0.09406(3)	0.14675(3)	0.02006(1)
		GdAA(67)	1/0	0.5	0.09492(3)	0.14356(3)	0.00527(1)
		GdAA(74)	1/0	0.5	0.09518(3)	0.1516(3)	0.00884(1)
		GdAA(82)	1/0	0.5	0.09466(4)	0.1511(4)	0.01402(2)
Au3/Al3	24g	GdAA(50)	0.321(4)/0.679(4)	0.2484(2)	0.0834(2)	0	0.0287(1)
		GdAA(55)	0.312(4)/0.688(4)	0.2479(2)	0.0830(2)	0	0.0327(1)
		GdAA(60)	0.289(3)/0.711(3)	0.2479(2)	0.0809(2)	0	0.0339(7)
		GdAA(67)	0.340(4)/0.660(4)	0.2467(2)	0.0765(2)	0	0.0309(9)
		GdAA(74)	0.510(4)/0.490(4)	0.2392(1)	0.0801(1)	0	0.0165(7)
		GdAA(82)	0.965(5)/0.035(5)	0.2384(9)	0.08985(9)	0	0.0241(6)
Au5/Al5	16f	GdAA(50)	0.958(5)/0.042(5)	0.15026(2)	0.15026(2)	0.15026(2)	0.01393(1)
		GdAA(55)	0.955(4)/0.045(4)	0.15025(2)	0.15025(2)	0.15025(2)	0.01889(1)
		GdAA(60)	1/0	0.15030(2)	0.15030(2)	0.15030(2)	0.01648(7)
		GdAA(67)	1/0	0.15002(2)	0.15002(2)	0.15002(2)	0.00861(7)
		GdAA(74)	1/0	0.15099(2)	0.15099(2)	0.15099(2)	0.01568(7)
		GdAA(82)	1/0	0.15278(3)	0.15278(3)	0.15278(3)	0.02147(1)
Au6/Al6	12e	GdAA(50)	0/1	0.5	0.1950(4)	0	0.0320(2)
		GdAA(55)	0/1	0.5	0.1954(4)	0	0.0345(2)
		GdAA(60)	0/1	0.5	0.1953(3)	0	0.0260(1)
		GdAA(67)	0/1	0.5	0.1979(4)	0	0.0079(1)
		GdAA(74)	0/1	0.5	0.1862(3)	0	0.0103(1)
		GdAA(82)	0.148(4)/0.852(4)	0.5	0.1884(2)	0	0.0110(8)
Au7/Al7	12d	GdAA(50)	0.432(5)/0.568(5)	0.4003(2)	0	0	0.0289(1)
		GdAA(55)	0.446(5)/0.554(5)	0.4001(2)	0	0	0.0335(1)
		GdAA(60)	0.513(4)/0.487(4)	0.3980(2)	0	0	0.0316(9)
		GdAA(67)	0.511(6)/0.489(6)	0.3965(3)	0	0	0.0257(1)
		GdAA(74)	0.984(5)/0.016(5)	0.4053(1)	0	0	0.0265(6)
		GdAA(82)	1/0	0.4069(1)	0	0	0.0241(6)
Al8	8c	GdAA(50)	1	0.25	0.25	0.25	0.0402(1)
		GdAA(55)	1	0.25	0.25	0.25	0.0436(1)
		GdAA(60)	1	0.25	0.25	0.25	0.0319(8)
		GdAA(67)	1	0.25	0.25	0.25	0.0085(7)
		GdAA(74)	1	0.25	0.25	0.25	0.0152(7)
		GdAA(82)	1	0.25	0.25	0.25	0.0255(1)
Au4/Al4	24g	GdAA(50)	0.378(12)/0.622(12)	0.0867(1)	0	0.0543(2)	0.071(5)
		GdAA(55)	0.378(9)/0.622 (9)	0.0863(2)	0	0.0537(2)	0.073(6)
		GdAA(60)	0.434(9)/0.566(9)	0.0894(5)	0	0.0521(7)	0.050(2)
		GdAA(67)	0.518(12)/0.482(12)	0.0898(7)	0	0.0527(8)	0.049(3)
		GdAA(74)	0.489(12)/0.511(12)	0.0735(7)	0	0.0702(7)	0.052(3)
		GdAA(82)	0.455(273)/0.212(273)	0.0790(3)	0	0.0520(5)	0.090(2)
Au9/Al9	16f	GdAA(82)	0.212(69)/0.121(69)	0.0530(3)	0.0530(3)	0.0530(3)	0.170(2)

**Figure 3:**
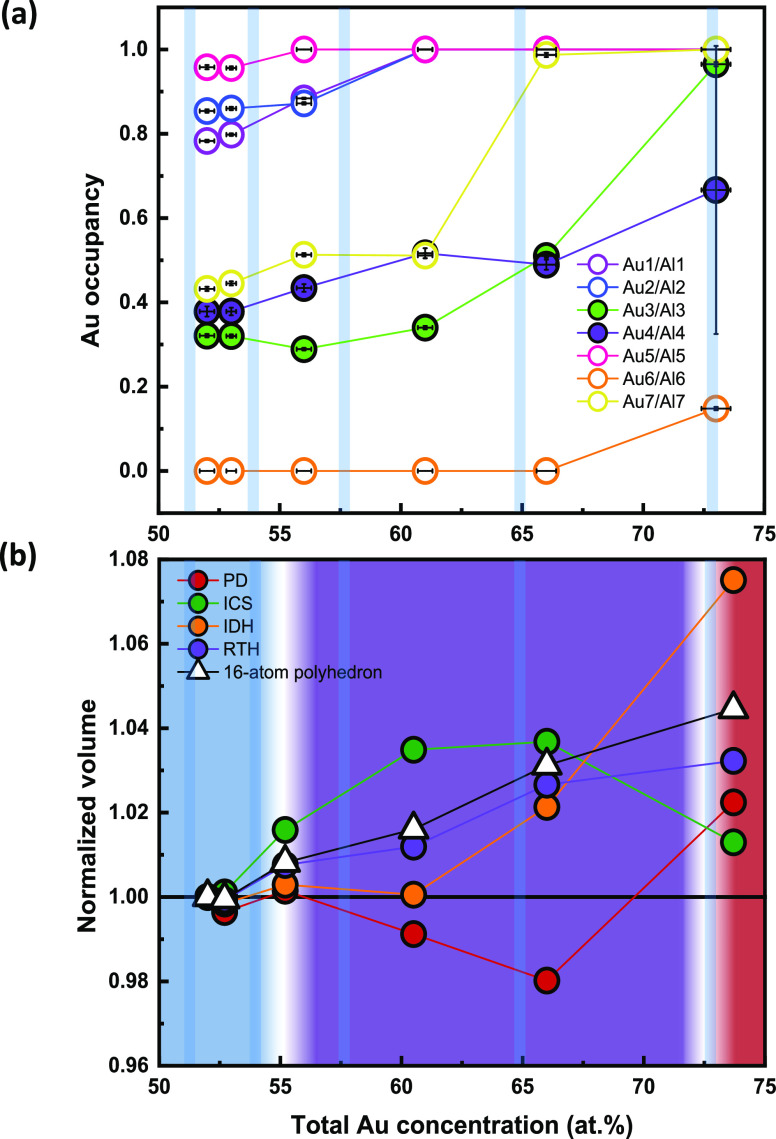
(a) Site occupancies and (b) normalized volume of polyhedra plotted as a function of the total Au concentration in the series of samples. In panel (a), each atomic position belongs to a different shell. The large error bar of the Au4/Al4 site for the highest Au sample arises from the addition of the extra position (Au9/Al9), cf. Table 3. In panel (b), the volumes of the polyhedra are normalized with respect to the values for the lowest Au concentration. The blue bars shown in both panels (a) and (b) are associated with inflection points of the lattice parameter trend and the FM-AFM change shown in Figure 1. Note that the triangle icon in panel (b) represents the volume of the 16-atom nearest neighbor polyhedron for Gd shown in Figure 1.

Figure 2 compares the crystal structures for the lowest and highest Au contents, Gd_14_Au_52_Al_34_ and Gd_14_Au_73_Al_13_, respectively. The structure of the Au-poor sample contains 7 mixed occupied positions (Au1/Al1, Au2/Al2, Au3/Al3, Au4/Al4, Au5/Al5, Au6/Al6, and Au7/Al7), which systematically reduce to two (Au3/Al3 and Au4/Al4) with increasing Au concentration *x* (see Figure 3a). Increasing the Au content implies also depletion of Al in the outermost RTH and IDH shells, i.e., a high Au concentration increases the chemical order.^4^ In addition, for the sample with the highest Au content (Gd_14_Au_73_Al_13_), the Al6 site (12e), belonging to the outermost RTH shell, starts to mix with Au. We will discuss occupancy variations in detail in the next section.

## Analysis of Occupancy Variations

The occupancy variation of the various sites as a function of Au concentration is shown in Figure 3a, where Al8 (8c) is a pure Al site for all investigated compositions and Au6/Al6 (12e) is essentially pure Al with a small Au occupancy at the largest *x*. This agrees with previous analyses of RE–Au–Al 1/1 AC structures (RE = Tm and Yb).^21,22^ In addition, the nonzero Au occupancy at the 12e site (RTH vertex position) for high Au concentrations (*x* = 73) agrees with the previous refinement of Gd_14_Au_73_Al_13_ by Ishikawa et al.^4^

The Au5/Al5 site (PD shell) is the first site to be fully occupied by Au at x=56. The Au1/Al1 site (which is present in both IDH and RTH shells) and Au2/Al2 (RTH shell) already show a high preference for Au in Au-poor samples (*x* < 60), and both become exclusively occupied by Au for *x* > 60. The Au3/Al3 site (PD shell) has a rather low and constant Au occupancy (around 30%) up to *x* = 61, beyond which *x* increases linearly to almost full Au occupancy at *x* = 73. The position Au7/Al7 (IDH shell) also exhibits a similar behavior, with a Au occupancy of around 45% up to *x* = 61, which increases abruptly to 100% for higher *x*. To summarize, as already reported by Ishikawa et al.,^4^ chemical order increases with increasing Au content as all mixed sites show an increase in the occupancy of Au. The interesting observation is that even the pure Al position 12e (Al6) can accept some Au in the sample with the highest Au content, Gd_14_Au_73_Al_13_. The position Au4/Al4 (and Au9/Al9) defining the tetrahedron at the center of the Tsai cluster is special. This tetrahedral unit breaks the icosahedral symmetry of the surrounding shells of an ideal Tsai cluster. As seen in Figure 3a, the occupancy changes only slightly from Au/Al = 40/60 to 60/40 for increasing *x*. However, the distribution of the electron density, as extracted from Fourier maps, changes significantly as a function of Au concentration (Figure S7a) and so does the shape of the refined ADPs ellipsoids (Figure S7b). The highest Au content sample has an additional position (Au9/Al9), which accounts for a triple split of electron density close to the 3-fold rotation axis^23^ (see Table 3). The sum of occupancies for the Au4/Al4 (and Au9/Al9) positions defining the cluster center adds up to four atoms regardless of the shape of the electron density.^23,24^ These electron densities need to be described with higher order anharmonic tensors in the refinement to reproduce their irregular shape. It is not really clear why the disorder at the cluster center is changed for Au-rich Gd_14_Au_73_Al_13_ (GdAA(82)). We note that a similar tetrahedral disorder situation was recently inferred for 1/1 AC Tb_15_Cd_65_Mg_20_.^25^

Deviations from Vegard’s law manifest as linear segments with different slopes separated by inflection points (Figure. 1). These inflection points typically coincide with points of full Au saturation for specific atomic sites (Figure 3a). Because these atomic sites have different point symmetries, their population by larger Au ions will induce local distortions that may affect the magnetic properties of the material. After reaching a saturation point, other positions in the structure are forced to accept higher amounts of Au as the total Au content in the sample continues to increase, causing an abrupt change in the linear slope of the cell parameter.

To understand the changes in magnetic properties, it is useful to elucidate all relevant structural events that take place as we systematically increase the total Au content. The first inflection point in Figure 1 takes place at *x* ≈ 51. Being the first measurement point in our set of investigated samples, we cannot see what change has taken place, as we have no structure with lower Au content to compare with, but the point can be clearly seen as distinguished in the data of Ishikawa et al.,^3^ both in the evolution of the cell parameter and the paramagnetic Curie–Weiss temperature. The second inflection point at *x* ≈ 53 coincides with both the saturation point of the atomic position Au5/Al5 (Figure 3a) and the border separating the SG from the FM regions (Figure 1). The third inflection point at *x* ≈ 58 coincides with the saturation points of the atomic positions Au1/Al1 and Au2/Al2 and may also be correlated with a change in the trend of the paramagnetic Curie–Weiss temperature. The fourth inflection point can be observed at *x* ≈ 65. At this composition, the Au7/Al7 position reaches full saturation.

### Volume of the Polyhedral Cluster Shells

Figure 3b depicts the variation of the polyhedral volumes for the different atomic shells as a function of the total Au concentration. In spite of a continuous expansion of the unit cell with increasing Au concentration, several atomic shells actually contract within certain ranges of Au concentration. The volume changes are nonlinear and have discontinuities that match the inflection points of the lattice parameter trend seen in Figure 1. Consequently, these volume discontinuities also correlate with the paramagnetic Curie–Weiss temperatures. In the following, we want to highlight the behavior of the PD, ICS, and IDH shells, showing decreasing volumes within certain ranges of Au concentration.

**Figure 4:**
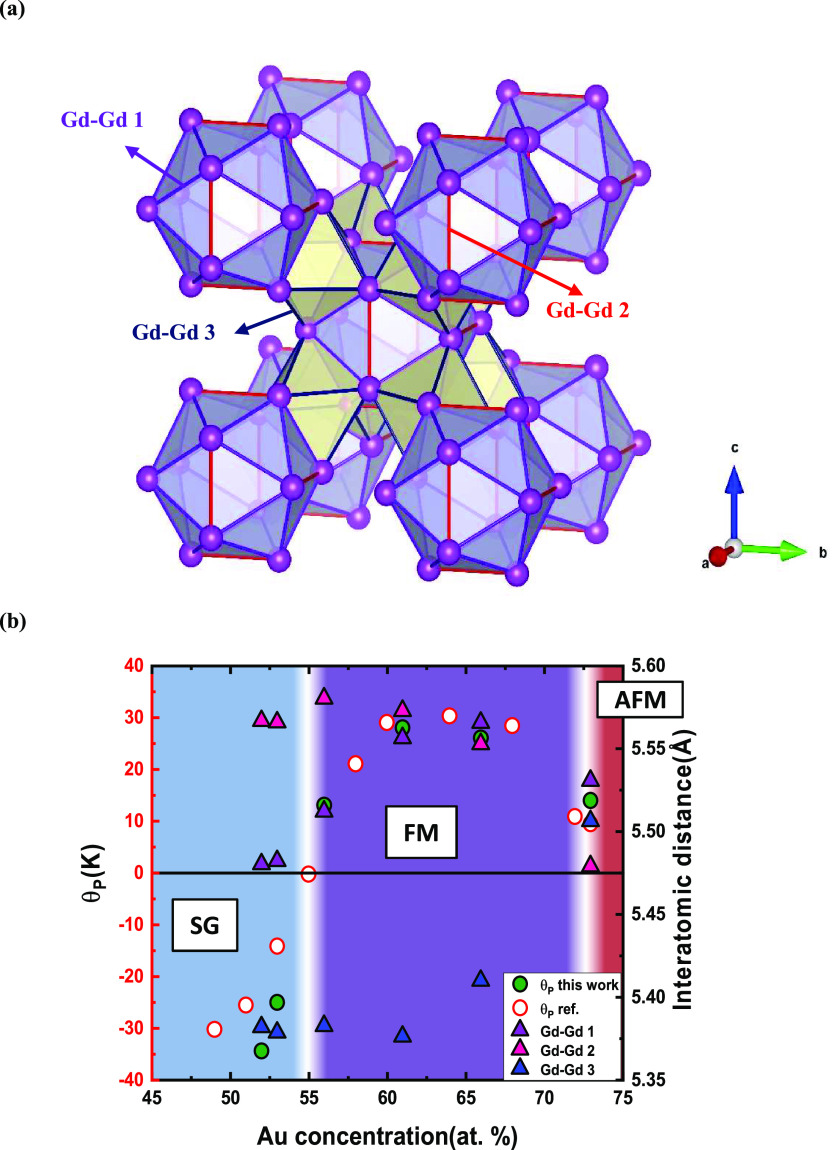
(a) Illustration of the ICS polyhedra in the 1/1 AC unit cell. It presents the geometrical correlation of three different Gd–Gd interatomic distances in the unit cell. The Gd–Gd_1 and Gd–Gd_2 interatomic distances appear in the intershell of the ICS shell (seen in purple and red, respectively), while the Gd–Gd_3 interatomic distance (blue) is the shortest Gd–Gd connection between adjacent icosahedra. This distance defined the edges of an octahedral junction (yellow) that connects the Gd icosahedra along the 3-fold directions. (b) Comparison of the magnetic phase diagram and Gd–Gd interatomic distances.

The Au5/Al5, Au1/Al1, Au7/Al7, and Au3/Al3 atomic sites are the main cause of the slope changes in the volume variations of the atomic shells, which take place at *x* ≈ 56, 61, and 65, respectively (see Figure 3a). At *x* ≈ 56, the Au5 atomic site reaches the saturation point and no longer contributes to the volume change of the PD shell. It implies that the volume of the PD shell is dominantly influenced by the occupancy of the Au3 site when *x* > 56. Interestingly, at *x* ≈ 56, there is simultaneously a discontinuity in the trend of the lattice parameter and the volume change of the PD shell, and *x* ≈ 56 represents the border where magnetism changes from SG to FM (see Figure 1). This indicates that the composition change causes systematic changes in both the structure and magnetic ordering. One possible explanation is the opposite volume change in the ICS and PD shells, releasing the space around the ICS shell and further reducing the geometric frustration by decreasing the differences in interatomic distances (*d*_(Gd–Gd_1)_ and *d*_(Gd–Gd_2),_ see Figure 4b) of the ICS. We will discuss interatomic distance within the ICS shell in detail in the next section.

Another discontinuity is reached at *x* ≈ 61 upon saturation of the Au1/Al1 site, which affects the slope in the volume of the IDH shell and also the trend in the paramagnetic Curie–Weiss temperature. At higher *x*, the IDH volume increases as the Au7/Al7 sites gradually reach full saturation. Notably, the volume changes of the PD and IDH shells for *x* > 61 have opposite signs in Figure 3b. This could give rise to relaxant geometrical frustration by causing changes in the Gd–Gd interatomic distances, as discussed in the next section. A third discontinuity is observed at *x* ≈ 65 in both the volume trends of the PD and ICS shells and the paramagnetic Curie–Weiss temperature, coinciding with lattice parameter inflection. The degree of volume increase in the IDH shell gradually exceeds the increase in the ICS as Au7/Al7 reaches saturation. In contrast, the PD shell shows the lowest volume since the occupancy change in the Au3 site is small. The last point to discuss is x ≈ 73. Here the volume of both the IDH and PD shells appears substantially increased as the Au3/Al3 site reaches nearly full saturation. Counterintuitively, the volume of the ICS shell is decreased. At this composition, the interatomic Gd–Gd distances within the ICS shell have a crossover point (see Figure 4b) where the magnetic behavior transforms effectively from FM to AFM (see Figure 1). This indicates that the interatomic distances in the ICS shell could be a key parameter that affects the magnetism of the Gd_14_Au_*x*_Al_86–*x*_ samples.

### Atomic Distance Variations in the Icosahedral Shell

As discussed above, the saturation of the various atomic sites takes place irregularly with increasing Au content. Therefore, the 1/1 AC structure does not expand uniformly, and Tsai cluster shells may actually contract with increasing Au content. Also, interatomic distances may change anisotropically. We compiled the relevant interatomic distances in all samples in Table 4.

**Table 4:** Interatomic Distances in the Different Atomic Shells

**interatomic distance (Å)**							
**shell**	**distance type**	**GdAA(50)**	**GdAA(55)**	**GdAA(60)**	**GdAA(67)**	**GdAA(74)**	**GdAA(82)**
disordered tetrahedron	Al4–Al4	1.5711(2)	1.5608(2)	1.6135(1)	1.6235(1)	1.4981(1)	1.4560(6)
	Al4–Al4	1.5926(4)	1.5747(4)	1.5286(2)	1.5481(2)	2.1655(1)	2.3389(6)
PD	Au3–Au3	2.4417(4)	2.4302(4)	2.3723(4)	2.2442(4)	2.3570(3)	2.6602(2)
	Au3–Au5	2.8035(2)	2.8009(2)	2.8191(2)	2.8356(2)	2.7799(1)	2.7548(9)
ICS(rare earth)	Gd–Gd_1	5.4799(7)	5.4818(4)	5.5118(6)	5.5562(7)	5.5655(4)	5.5304(7)
	Gd–Gd_2	5.5666(8)	5.5658(6)	5.5802(7)	5.5727(9)	5.5526(6)	5.4787(1)
	Gd–Gd_3	5.3813(7)	5.3780(3)	5.3820(6)	5.3756(7)	5.4094(4)	5.5060(7)
IDH	Au1–Au1	4.2856(6)	4.2835(6)	4.2983(5)	4.2852(4)	4.3028(4)	4.3378(7)
	Au1–Au1	3.1068(6)	3.1031(6)	3.0885(5)	3.0571(4)	3.1074(4)	3.1996(7)
	Au1–Au7	3.3622(9)	3.3616(9)	3.3687(8)	3.3982(1)	3.4225(5)	3.4951(7)
RTH	Au1–Au1	4.2856(6)	4.2835(6)	4.2983(5)	4.2852(4)	4.3028(4)	4.3378(7)
	Au2–Al6	2.6170(3)	2.6197(3)	2.6153(3)	2.5949(4)	2.6048(2)	2.6319(2)
	Au2–Al6	2.6839(5)	2.6783(3)	2.6968(2)	2.7140(3)	2.7706(2)	2.7588(2)

In this section, we want to especially highlight the interatomic Gd–Gd distances, which are the important parameter when considering the Ruderman–Kittel–Kasuya–Yosida (RKKY) interaction for mediating the localized magnetic moments on the Gd atoms.^9^ There are two nearest neighbor interatomic distances within an ICS shell (Gd–Gd_1 and Gd–Gd_2) and one between ICS shells (Gd–Gd_3). Gd–Gd_3 and Gd–Gd_1 distances together define a network of corner-connected (distorted) octahedra (see Figure 4a). Gd–Gd_2 distances are situated on symmetry planes/perpendicular to 2-fold rotational axes, and Gd–Gd_3 distances align along the 3-fold rotational axes. The three kinds of distances are in the range of 5.35 to 5.6 Å (cf. Table 4).

Initially, the ICS shell expands up to *x* ≈ 61 (see Figure 3b). This is accompanied by an increase in *d*_(Gd–Gd_1)_ from 5.48 to 5.56 Å, whereas *d*_(Gd–Gd_2)_ and also *d*_(Gd–Gd_3)_ vary insignificantly (5.57–5.58 and 5.38 Å, respectively), cf. Table 4. We conjecture that this feature relates to the increased Au occupancy on the site Au1/Al1 being part of the IDH shell (Figure 3a and Figure S8). Interestingly, we notice that the variation of the Gd–Gd_1 distance vs total Au concentration shows a striking correlation to the variation in the paramagnetic Curie–Weiss temperature (Figure 4b), which in turn may imply that *d*_(Gd–Gd_1)_ plays a crucial role in the nature of the magnetic transition. For *x* > 65, concomitant with the full saturation of the site Au7/Al7 and nearly full saturation of the site Au3/Al3, *d*_(Gd–Gd_2)_ decreases and *d*_(Gd–Gd_3)_ increases significantly (Figure S8). At these high Au concentrations, Gd atoms are almost exclusively coordinated by Au atoms (cf. Figure 1). The Gd–Gd_3 distance is always the shortest interatomic distance until *x* ≈ 73, which correlates with the transition to AFM. We conclude the analysis of Gd–Gd distances by noting a phenomenological correlation with the various magnetic regions established for Gd_14_Au_*x*_Al_86–*x*_. In the spin glass region, *d*_(Gd–Gd_1)_ < *d*_(Gd–Gd_2)_. In the FM region, *d*_(Gd–Gd_1)_ ≈ *d*_(Gd–Gd_2)_. In the AFM region, *d*_(Gd–Gd_1)_ > *d*_(Gd–Gd_2)_. From a structural point of view, the variation of the Au concentration not only changes the *e*/*a* ratio but also infers nonlinear changes to the atomic structure, affecting the distance between the moment bearing Gd atoms, which ultimately should affect the magnetic structure. Yet if the RKKY interaction is the dominant spin interaction, the *e*/*a* ratio will be the decisive parameter for explaining composition-driven magnetic property changes in Tsai-type AC systems.^26^

## Conclusions

We have reported crystal structure refinements of a collection of 6 different Gd_14_Au_*x*_Al_86–*x*_ 1/1 AC samples with varying Au/Al compositions (*x* = 52, 53, 56, 61, 66, and 73). With increasing Au concentration, we observe that Au tends to occupy Au/Al mixed sites preferentially from the outer to inner shells of the Tsai clusters as the Au concentration increases. Among the different sites defining shells, the Au3/Al3 site located in the PD shell appears the hardest to saturate as a pure Au site. Distinct scenarios of chemical Au/Al ordering can be distinguished (*x* = 53, 58, and 65) that correlate with inflection points in the lattice parameter trend. In addition, it was found that Gd–Gd nearest neighbor distances vary irregularly with Au concentration. Yet Gd–Gd distance variations correlate closely with the magnetic transitions observed across *x*. A most important observation is the variation of the Gd–Gd_1 distance, as it almost perfectly mirrors the trend of the paramagnetic Curie–Weiss temperature with increasing Au concentration.

The nonrandom chemical disorder may be the origin of the nonlinear trends and explain the deviations from Vegard’s law. Importantly, varying the Au concentration not only changes the *e*/*a* ratio but also infers nonlinear changes to the atomic structure, affecting the interatomic distance between Gd atoms that ultimately may affect the magnetic structure. The underlying composition-triggered local distortions of the ICS shell may be explained by an increase in negative chemical pressure on the Gd atoms since with increasing Au concentration, the volume of the 16-atom nearest neighbor polyhedron increases.^27^ To answer the question of whether the structural changes or the change in *e*/*a* drives the different magnetic ordering, one may think of performing bulk magnetic measurements under physical pressure. The application of physical pressure on Gd_14_Au_73_Al_13_ and Gd_14_Au_61_Al_25_ (or Gd_14_Au_56_Al_30_) may lead to AFM-FM and FM-SG transitions, respectively. Such experiments would lead to a better understanding of the correlation between the crystal structure and magnetic order in ACs, and they may also provide a platform to find stable and long-range magnetic order of the QCs.

## Supporting Information Available

DSC traces of reaction mixtures, images of crystalline products, reciprocal space layers with superlattice reflections with respect to the basic cubic cell for GdAA(50), PXRD patterns for all samples, DSC traces for Gd_14_Au_*x*_Al_86–*x*_ 1/1 AC specimens and PXRD patterns before and after for DCS runs, electron density isosurface plots of the cluster center for GdAA(*xx*), compilation of results from magnetic properties measurements, temperature dependence of the magnetic susceptibility and inverse magnetic susceptibility, and Gd–Gd interatomic distances and occupancies of Au/Al atomic sites being part of the PD and IDH shells as a function of the total Au concentration (PDF)

## Accession Codes

CCDC 2268534–2268539 contain the supplementary crystallographic data for this paper. These data can be obtained free of charge via www.ccdc.cam.ac.uk/data_request/cif, or by emailing data_request@ccdc.cam.ac.uk, or by contacting The Cambridge Crystallographic Data Centre, 12 Union Road, Cambridge CB2 1EZ, UK; fax: +44 1223 336033.

## Abbreviations

i-QCs: icosahedral quasicrystals
ACs: quasicrystal approximants
RE: rare-earth elements
PXRD: powder X-ray diffraction
SCXRD: single-crystal X-ray diffraction
DSC: differential scanning calorimetry
SEM: scanning electron microscopy
EDX: energy dispersive X-ray
GdAA: Gd–Au–Al
θ_p_: paramagnetic Curie–Weiss temperature
SG: spin glass
FM: ferromagnetic
AFM: antiferromagnetic
*e*/*a*: valence electron concentration
RKKY: Ruderman–Kittel–Kasuya–Yosida interactions
PD: pentagonal dodecahedral
ICS: icosahedral
IDH: icosa-dodecahedral
RTH: rhombic triacontahedron
ADPs: atomic displacement parameters
ZFC: zero-field cooled
FC: field cooled
