# Supplementary material for: Structural Analysis of the Gd–Au–Al 1/1 Quasicrystal Approximant Phase across Its Composition-Driven Magnetic Property Changes
Source: Inorg Chem. 2023 Aug 29;62(36):14668–77. doi: 10.1021/acs.inorgchem.3c01967 (PMC10498487; doi:10.1021/acs.inorgchem.3c01967)

Supporting Information for

Structural analysis of the Gd-Au-Al 1/1 quasicrystal approximant phase across its composition-driven magnetic property changes.

Yu-Chin Huang[1], Ulrich Häussermann[2], Girma H. Gebresenbut[1], Fernand Denoel[3], Cesar Pay Gómez[1]*

[1]Department of Chemistry-Ångström laboratory, Uppsala University, 751 21 Uppsala, Sweden
[2]Department of Materials and Environmental Chemistry, Stockholm university, 106 91 Stockholm, Sweden
[3]Department of Materials Science and Engineering, Uppsala University, Box 35, 751 03 Uppsala, Sweden

Quasicrystal approximant, crystal structure, e/a ratio, magnetism, spin glass, ferromagnetic, antiferromagnetic, chemical disorder.

*yu_chin.huang@kemi.uu.se

Figure S1. DSC cooling traces for reaction mixtures with indicated nominal compositions $Gd_8(Au_zAl_{100-z})_{92}$. The liquidus temperature at which precipitation of crystalline 1/1 AC $Gd_{14}Au_xAl_{86-x}$ phase occurs (exothermic event) is in the range 800-950°C.

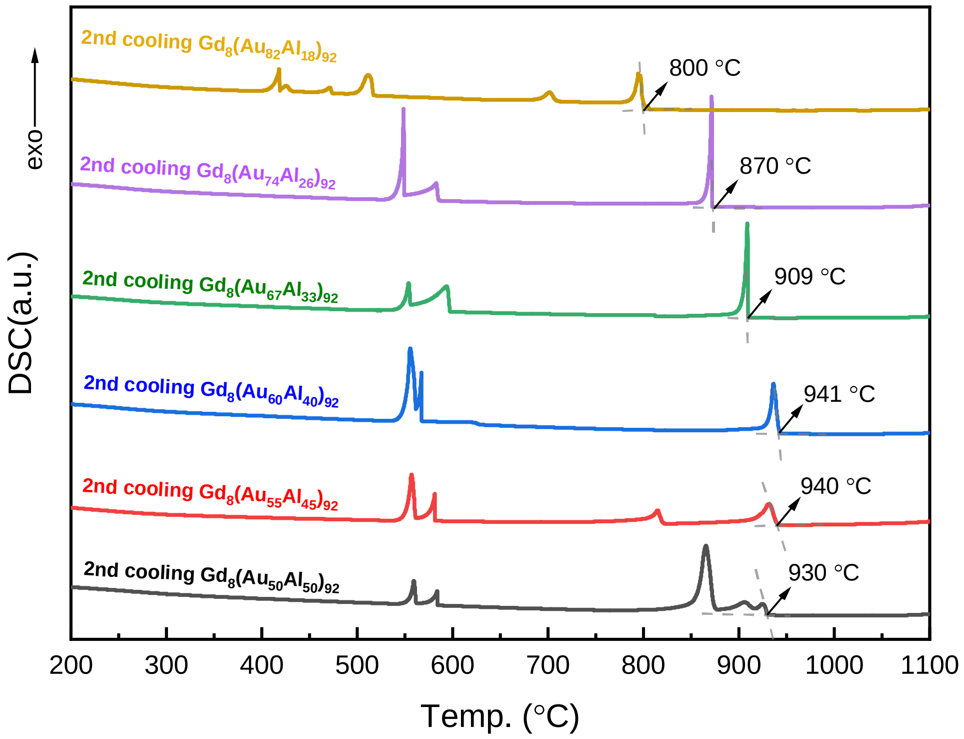

Figure S2. Optical microscope images of the mm-sized crystals obtained from solution growth synthesis with indicated nominal compositions.

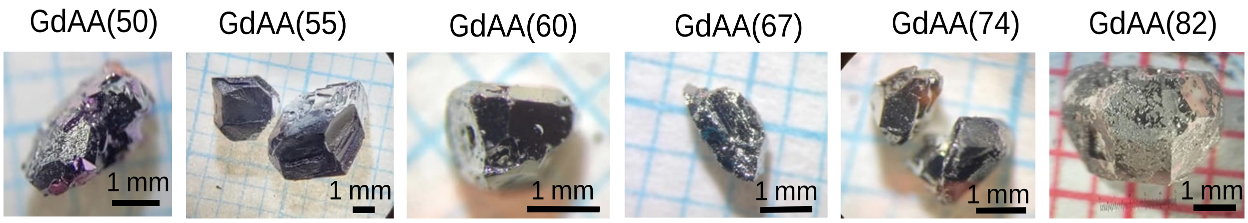

Figure S3. Reciprocal space layers of the GdAA(50) sample. The *hk0*, *hk0.5*, and *hk1* sections with respect to the basic 1/1 AC cubic cell are displayed. The superstructure reflections appearing between the main reflections, have been marked with red arrows in the *hk0* and *hk1* sections. The *hk0.5* section only contains superstructure reflections.

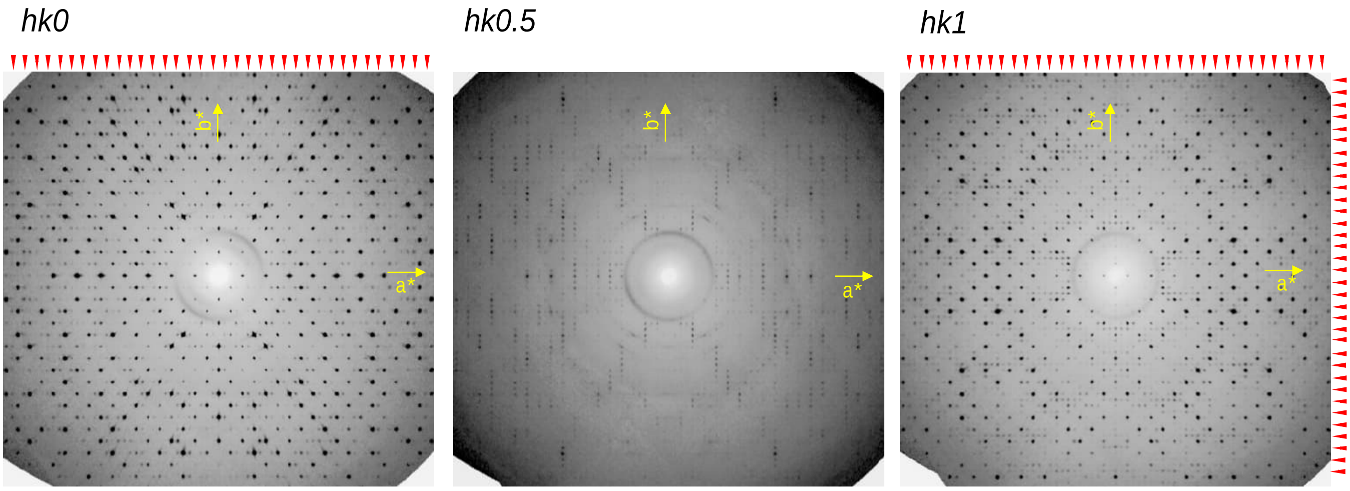

Figure S4. Powder X-ray diffraction data of $Gd_{14}Au_xAl_{86-x}$ samples with refined composition indicated. For accurate lattice parameter determination an internal Si standard was admixed. The x=52 sample showed parasitic peaks from residual flux (*f*). The systematic peak shift toward low 2θ angle implies that the lattice parameter expands as the Au concentration increases.

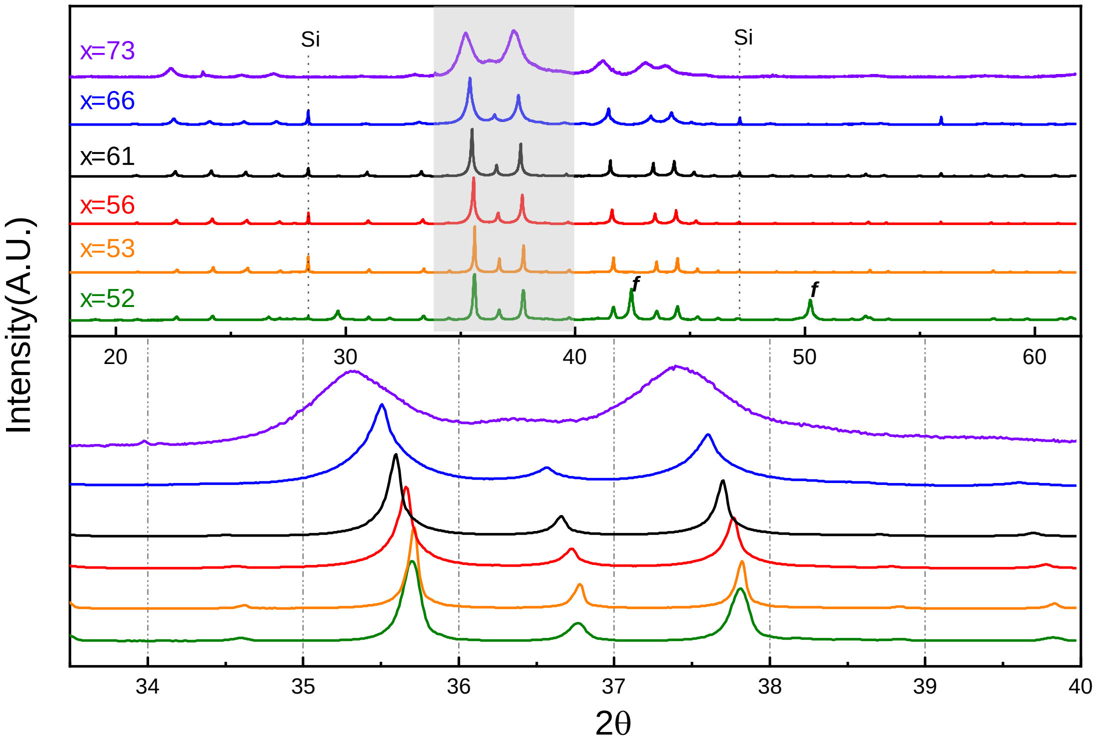

Figure S5. (a)-(f) DSC traces of $Gd_{14}Au_xAl_{86-x}$ 1/1 AC specimens (left) and PXRD patterns before (black) and after (red) the DSC measurements (right).

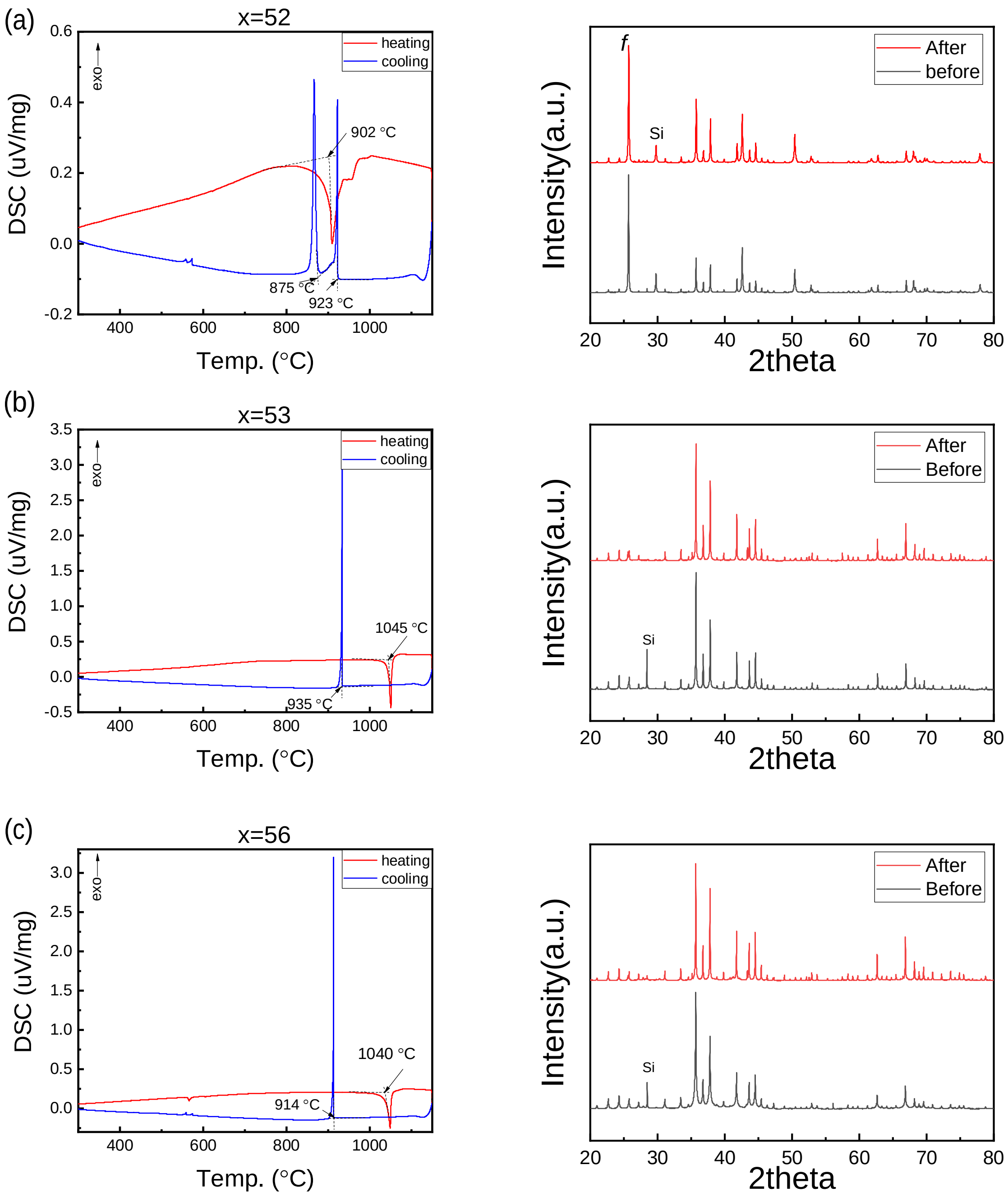

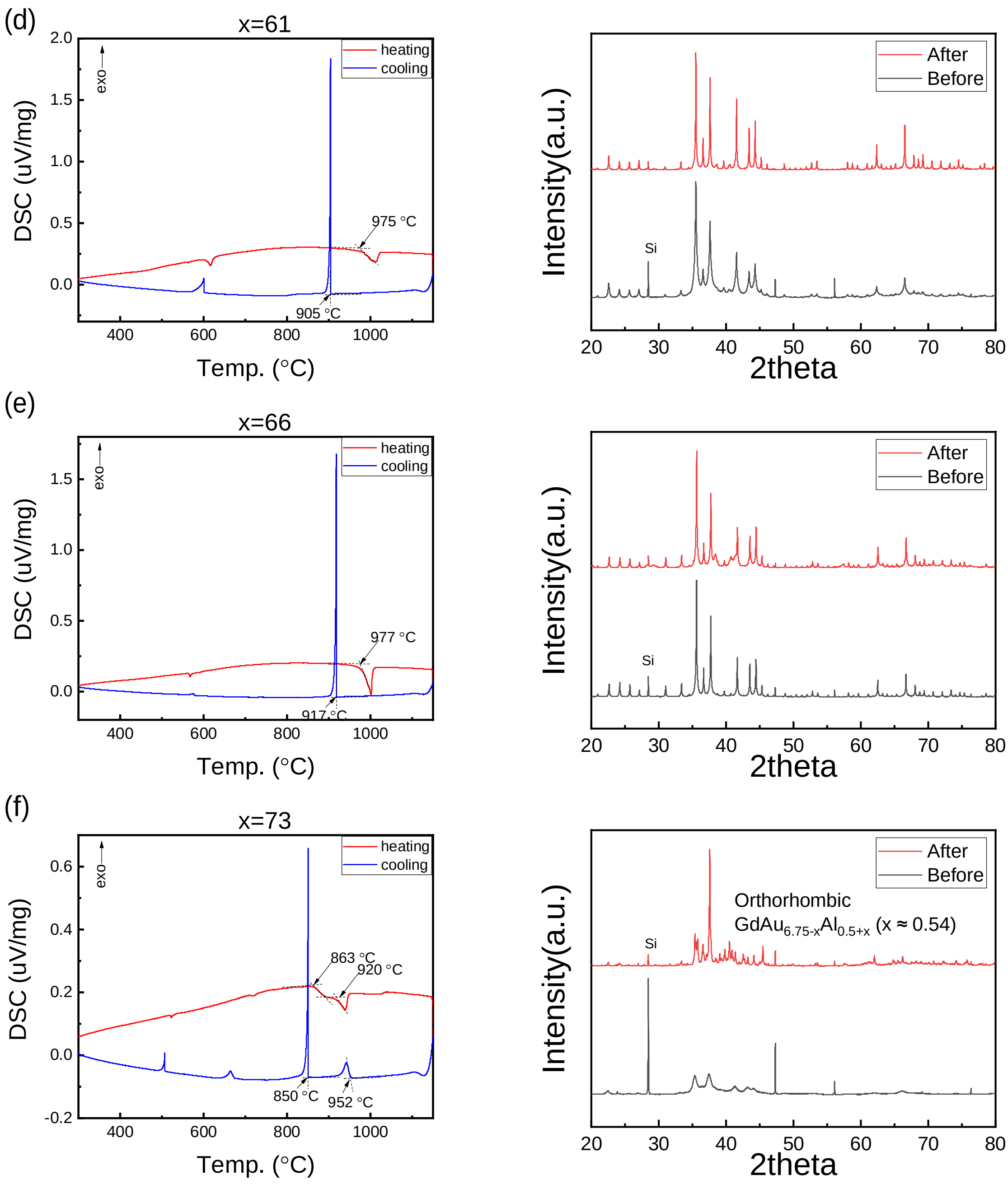

Table S1. The magnetic properties result

Sample	Composition(at. %)	Transition temperature (T_c, T_N or T_f)	p_{eff} (μB)	θ_p (K)	$\lvert\theta_p\rvert/T_m$*	Magnetic behavior
GdAA(50)	$Gd_{14.0(2)}$ $Au_{52.0(3)}$ $Al_{34.0(4)}$	3 K	8.0	-34.6	11.53	SG
GdAA(55)	$Gd_{14.0(2)}$ $Au_{53.0(2)}$ $Al_{33.0(2)}$	3.4 K	8.5	-25.2	7.41	SG
GdAA(60)	$Gd_{13.9(4)}$ $Au_{56.0(3)}$ $Al_{30.2(5)}$	12 K	8.2	13	1.08	FM
GdAA(67)	$Gd_{13.8(4)}$ $Au_{61.4(3)}$ $Al_{24.8(6)}$	27 K	8.2	25.5	0.94	FM
GdAA(74)	$Gd_{13.6(3)}$ $Au_{66.4(4)}$ $Al_{20.0(4)}$	26 K	8.4	26	1	FM
GdAA(82)	$Gd_{13.6(2)}$ $Au_{73.1(6)}$ $Al_{13.3(6)}$	8 K	7.6	13.9	1.74	AFM

*The observed magnetic transition or spin freezing obtained from M(T) measurements under low field.

Figure S6. (a) Temperature dependence of the magnetic susceptibility and (b) Temperature dependence of the inverse magnetic susceptibility for $Gd_{14}Au_xAl_{86-x}$ samples. The antiferromagnetic sample with Au concentration x = 73 shows a relatively large negative background, which is associated with the presence of a small FM impurity phase and the intrinsically low magnetization of the sample.

(a)

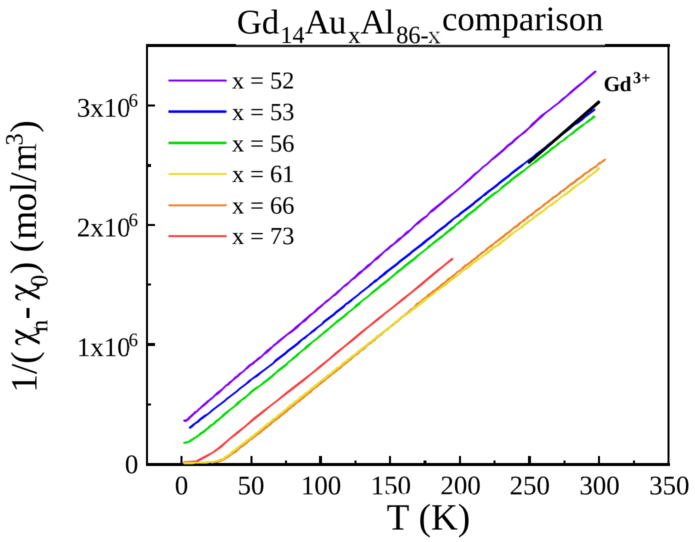

(b)

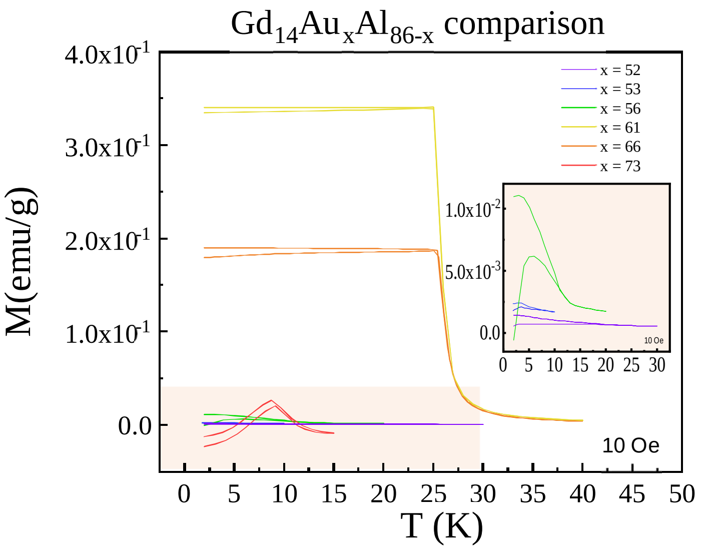

Figure S7. Electron density iso-surfaces at a 20 eÅ^{-3} level near the Tsai cluster center (a) and displacement ellipsoids at the 50% probability level for atomic positions describing the disordered tetrahedra at the cluster centers (b). As the Au concentration increases, the shape and size of the electron density changes, especially the disordered tetrahedron shrinks in GdAA(82). There is an extra split position, which shows an additional electron density appearing close to the 3-fold axis described by the Au9/Al9 position.

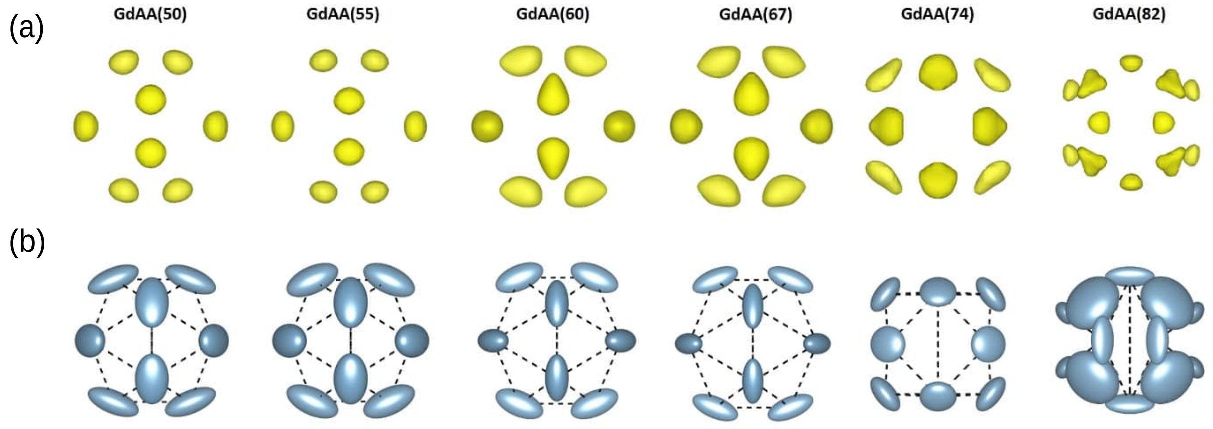

Figure S8. Gd-Gd interatomic distances and occupancies of Au/Al atomic sites being part of the PD and IDH shells as a function of the total Au concentration.

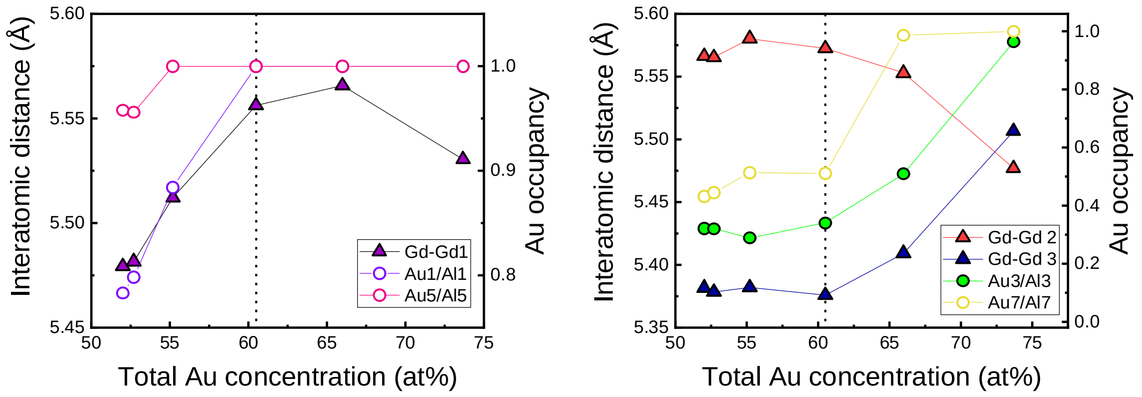

Supplement: Supplementary file 1 — ic3c01967_si_001.pdf [file ic3c01967_si_001.pdf]
